# The Sanitary Backsliding of Cuba

**Published:** 1905-03

**Authors:** 


### THE SANITARY BACKSLIDING OF CUBA.

There have been for some time persistent rumors of the reappearance of yellow fever in several of the Cuban towns, and three cases have been officially reported within the past month by the U. S. Public Health and Marine Hospital Service. These three cases were not imported from Mexico, but are said to have originated at Punta de Sal in Santiago province. If this is true—and a dispatch from Havana says that Dr. Finlay, chief of the Cuban Board of Health, admits that at least one case originated on the island—it is a matter of grave importance. The disease can not arise *de novo*, and if one case has originated at Punta de Sal there must have been a previous case, and possibly, even probably, a series of cases. This fact, taken with the known filthy condition of Santiago and other towns in the eastern end of the island is one to cause concern to this country. According to the report of the U. S. Chargé d'Affaires in Cuba, the streets of Santiago are in such a condition as to be most favorable to the breeding of disease germs, and particularly those of yellow fever. It is stated that there is practically no drainage, and that water is allowed to stand in the streets for days at a time. The press of Havana confirm these reports, one paper, *The Discussión*, stating that it is an undoubted fact that sanitation in nearly all the towns of the island is at such a low level that the public health is in danger. The paper declares that this condition of affairs is a blot on the republic and a shame to the Cuban people.

In consequence of these reports the State Department at Washington has called the attention of the Cuban Government to the necessity of rigid sanitary control of the situation in the island. This action of our government finds warrant in the sanitary article of the "Platt Amendment," which provides that "the Government of Cuba will execute and as far as necessary extend the plans already devised, or other plans to be mutually agreed upon for the sanitation of the cities of the island to the end that a recurrence of epidemic and infectious diseases may be prevented, thereby assuring protection to the people and commerce of Cuba, as well as to the commerce of the Southern ports of the United States and the people residing therein."

Perhaps this reminder from Washington and the near approach of the annual meeting of the Public Health Association in Havana will give the needed jog to the Cuban health authorities, who are thoroughly capable but have to struggle against the unsanitary traditions of Spanish officialdom.—*Medical Record.*

---

A school teacher boxed the ears of a pupil a few days ago. The boy told his mother, and the next day the teacher received the following note: "Nature has provided a proper place for the punishment of a boy, and it is not his ear. I will thank you to use it hereafter."

